# Supplementary material for: Hard Carbon Embedded with FeSiAl Flakes for Improved Microwave Absorption Properties
Source: Materials (Basel). 2022 Sep 1;15(17):6068. doi: 10.3390/ma15176068 (PMC9457624; doi:10.3390/ma15176068)
Supplement: Supplementary file 1 [file materials-15-06068-s001.zip › materials-1868698-supplementary.pdf]

Supporting Information

Hard carbon embedded with FeSiAl flakes for improved microwave absorption properties

Xiaogang Sun [1,2], Yi Liu[1], Daitao Kuang[3,*], Jun Lu[1], Junyi Yang[1], Xiaomin Peng[1,2,*], and Anru Wu[1,2]

1 College of Mechanical Engineering, Hunan Institute of Engineering, Xiangtan 411104, China;

2 Hunan Engineering Research Center of New Energy Vehicle Lightweight, Xiangtan 411104, China

3 School of Computational Science and Electronics, Hunan Institute of Engineering, Xiangtan 411104, China

* Correspondence: 806724093@qq.com (D. K.), xmpeng@hnie.edu.cn (X. P.)

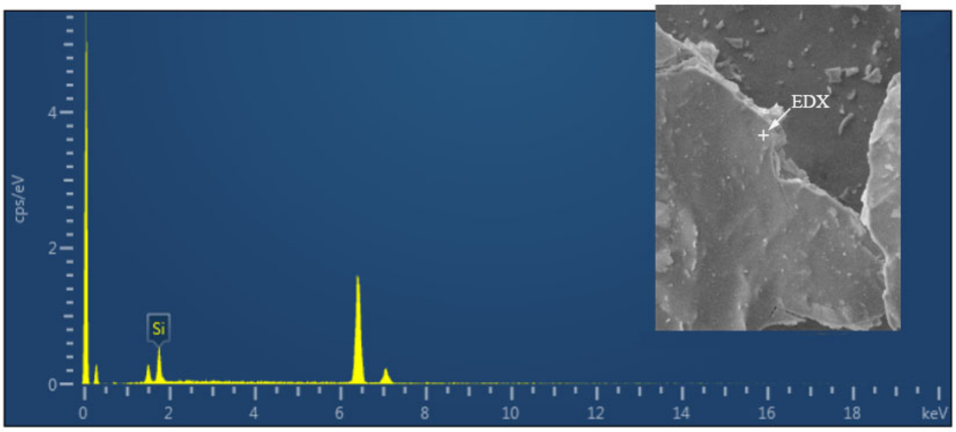

Figure S1. EDX analysis of the FeSiAl@HC sample (S3).

Elt.	Line	Wt %	Wt% sigma
Fe	Kα	86.59	0.68
Al	Kα	5.05	0.48
Si	Kα	8.36	0.52
Total		100.00	

Table S1. Element composition of FeSiAl flakes from EDS.

The EDX result revealed that the tested composition of FeSiAl flakes in the HC matrix is close to that of the raw material FeSiAl flakes (Si 9.8wt%, Al 5.6wt%, and Fe 84.6wt%).

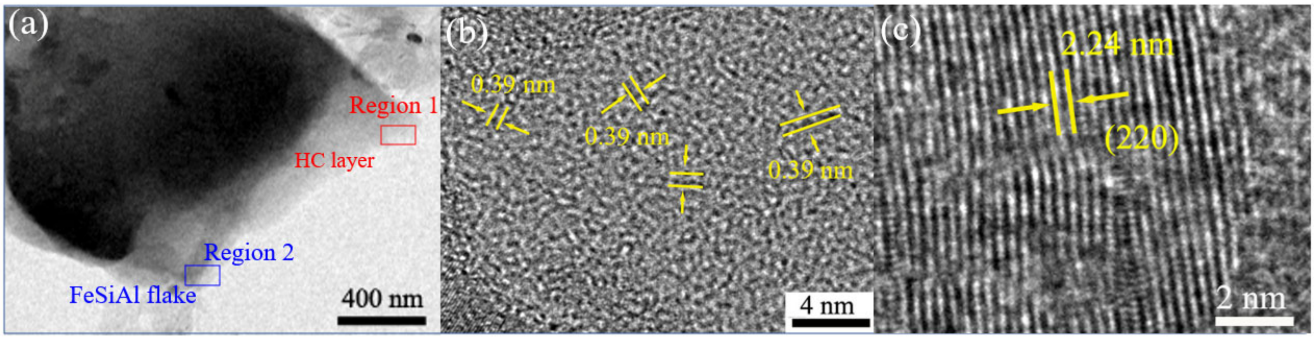

Figure S2. TEM image of S3 (a); High-resolution TEM image of the HC layer in region 1(b); High-resolution TEM image of a FeSiAl flake in region 2(c).

Figure S2 presents the typical TEM images of the as-fabricated FeSiAl@HC. As shown in Figure S2(a), it can be observed a FeSiAl flake was covered by a HC layer. Two regions located at the edge of FeSiAl@HC were selected for the high-resolution characterizations to confirm this result. High resolution TEM image in region 1 was shown in Figure S2(b). The lattice fringes of 0.39 nm well correspond to those values of 0.38–0.41 nm for HC, but obviously greater than those of 0.34–0.36 nm for graphite [15]. The high-resolution TEM image in region 2 was shown in Figure S2(c). The lattice fringe of 2.24 nm well consistence with (220) lattice spacing of FeSiAl.

[15] Liu L, Kuang D, Hou L, Luo H, Deng L and Wang S 2021 Synthesis and microwave absorption performance of layered hard carbon embedded with ZnO nanoparticles *J. Alloys Compd* 162677

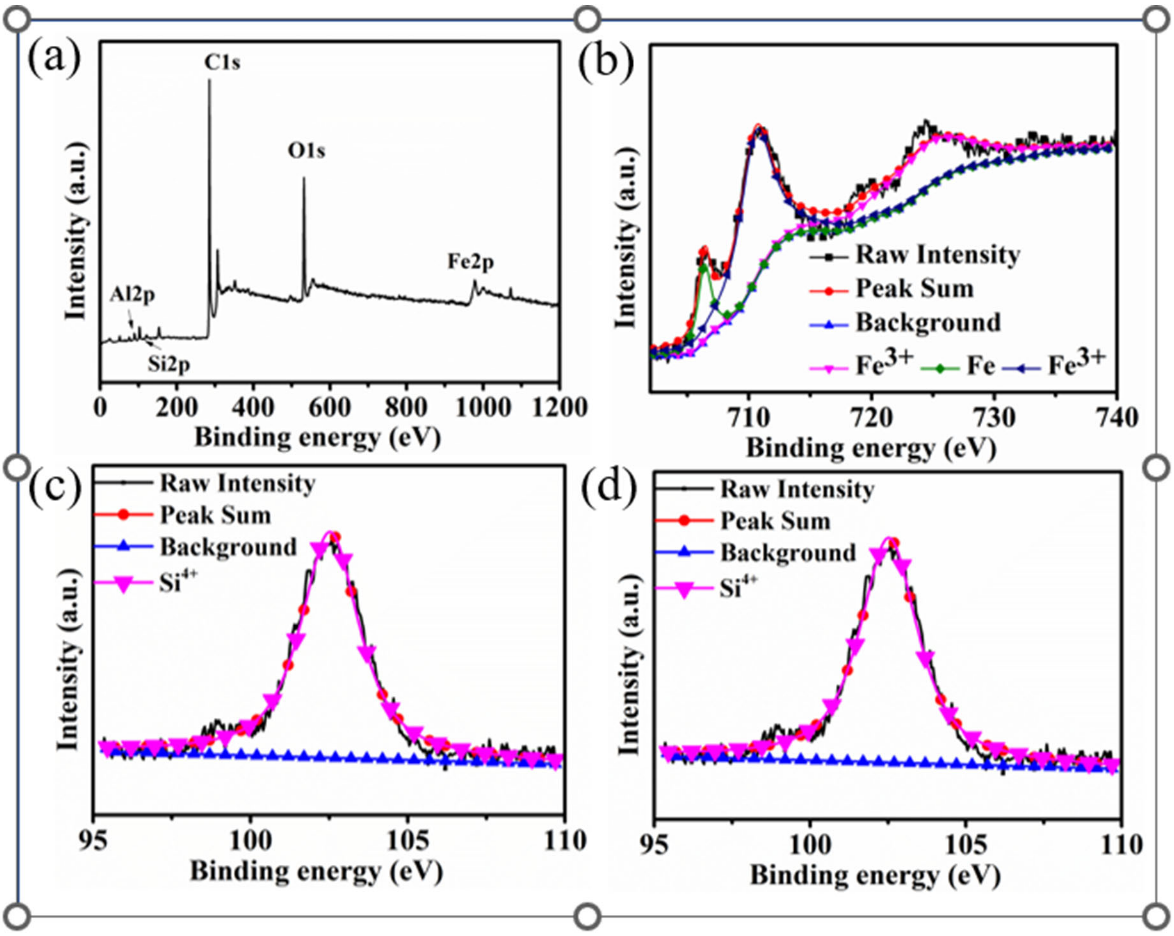

Figure S3. Wide scan XPS spectra of S3(a); Fe2p spectrum (b), Si2p (c)and Al2p(d) spectrum of S3.

Figure S3 shows the typical XPS spectra of the as-fabricated FeSiAl@HC. Figure S3(a) shows the survey spectrum of FeSiAl@HC, indicating the existence of Fe2p, Si2p, Al2p, C1s and O1s. O1s should come from the physisorbed water of in the sample [1, 2]. The high resolution Fe2p of FeSiAl@HC (Figure S3(b)) was fitted by deconvolution peaks at ~ 706.8, 711.6, and 725.2 eV, which relate to the $Fe2p_{3/2}$ of Fe metal, $Fe2p_{3/2}$ of Fe^{3+} and satellite feature of Fe^{3+}, respectively [3]. The existence of Fe can be attributed to the reduction of FeSiAl by the HC. Figure **(c) and (d) exhibit the high resolution Si2p and Al2p, which correspond to Si^{4+} and Al^{3+} of FeSiAl, respectively. Therefore, XPS results validate the formation of FeSiAl@HC.

[32] Kuang D, Hou L, Wang S, Luo H, Deng L, He J and Song M 2019 Facile synthesis of Fe/Fe_3C-C core-shell nanoparticles as a high-efficiency microwave absorber *Appl. Surf. Sci.* **493** 1083-9

[33] Kuang D, Hou L, Wang S, Luo H, Deng L, Mead J L, Huang H and Song M 2019 Large-scale synthesis and outstanding microwave absorption properties of carbon nanotubes coated by extremely small FeCo-C core-shell

nanoparticles *Carbon* **153** 52-61

[34] Kuang D, Wang S, Hou L, Luo H, Deng L, Song M, He J and Huang H 2020 Facile synthesis and influences of Fe/Ni ratio on the microwave absorption performance of ultra-small FeNi-C core-shell nanoparticles *MRS Bulletin* **126** 110837
